# A *de novo* triplication on 2q22.3 including the entire *ZEB2* gene associated with global developmental delay, multiple congenital anomalies and behavioral abnormalities

**DOI:** 10.1186/s13039-015-0206-8

**Published:** 2015-12-23

**Authors:** Haiming Yuan, Lina Zhang, Mengfan Chen, Junping Zhu, Zhe Meng, Liyang Liang

**Affiliations:** Sun Yat-Sen Memorial Hospital, Sun Yat-Sen University, Guangzhou, 510120, Guangdong China; Guangzhou kingmed center for clinical laboratory Co., Ltd, Guangzhou, 510330, Guangdong China; KingMed School of Laboratory Medicine Guangzhou Medical University, Guangzhou, 510330, Guangdong China

**Keywords:** Mowat-Wilson syndrome, Distinctive facial features, Intellectual disability, Developmental delay, Congenital anomalies, Behavioral abnormalities, *ZEB2*-triplication

## Abstract

**Background:**

Mowat-Wilson syndrome (MWS) is a genetic condition characterized by distinctive facial features, moderate to severe intellectual disability, developmental delay and multiple congenital anomalies. MWS is caused by heterozygous mutations or deletions of the *ZEB2* gene located on chromosome 2q22.3. At present, over 190 cases with mutations and deletions involving the *ZEB2* gene have been reported, but triplication or duplication of reciprocal region of Mowat-Wilson syndrome has never been reported.

**Case Presentation:**

Here we report a 2-year-2-month-old boy carrying a *de novo* 2.9 Mb complex copy number gain at 2q22.3 involving triplication of *ZEB2* gene. The boy is characterized by intrauterine growth retardation, hypotonia, cognitive impairment, multiple congenital anomalies and behavioral abnormalities.

**Conclusion:**

This case provides evidence that triplication of *ZEB2* gene may be clinical significance and *ZEB2* gene is likely to be a dosage sensitive gene.

## Background

Mowat-Wilson syndrome (MWS; OMIM# 235730) is an autosomal dominant genetic syndrome with multiple congenital anomalies. MWS is characterized by distinctive facial features, epilepsy, moderate to severe intellectual disability, global developmental delay, and congenital anomalies including agenesis of the corpus callosum, Hirschsprung disease, genitourinary anomalies, hypospadias, congenital heart disease, short stature and hypotonia [1–6]. MWS individuals display behavior problems including a happy affect and sociable demeanor, repetitive behaviors, pain insensitivity and a high rate of oral behaviors [7]. Eye abnormalities and craniosynostosis are rare features of this syndrome [8–10]. Eye abnormalities include iris/retinal colobomas, atrophy or absence of the optic nerve, hyphema, and deep refraction troubles, sometimes leading to severe visual consequences [8]. The syndrome is caused by heterozygous deletions or mutations of *ZEB2* (OMIM# 605802) gene located on chromosome 2q22.3. So far, more than 190 individuals with MWS have been described, who result from more than 100 different mutations or deletions of *ZEB2* gene. However, no obvious genotype-phenotype correlation was observed unless MWS patients carrying large deletions presented with more severe conditions, which may be the effect of continuous genes deletion [11–14]. Currently, no clinical presentations of patients with *ZEB2* copy number gain have been reported. Here, we report the first case of a *de novo* 2.9 Mb copy number gain at 2q22.3 involving triplication of the entire *ZEB2* gene detected by chromosomal microarray analysis (CMA). This case suggests that *ZEB2* gene is likely to be a dosage sensitive gene.

## Case presentation

The proband was the first child of healthy unrelated parents and family history was unremarkable. Intrauterine growth retardation was noticed by ultrasound examination at 7 months of pregnancy. He was born by vaginal delivery at 38 weeks of gestation. Birth weight was 3.0 kg (20.3 %), length 48 cm (8.5 %) and head circumference 32 cm (1.7 %). Apgar scores were all 9. He had severe hypotonia. No feeding difficulty was noted at all times. The development milestones were delayed: he raised his head at 4 months of age, sat alone at 8 months and walked without assistance at 1 year 8 months. Language development was significantly delayed.

**Fig. 1:**
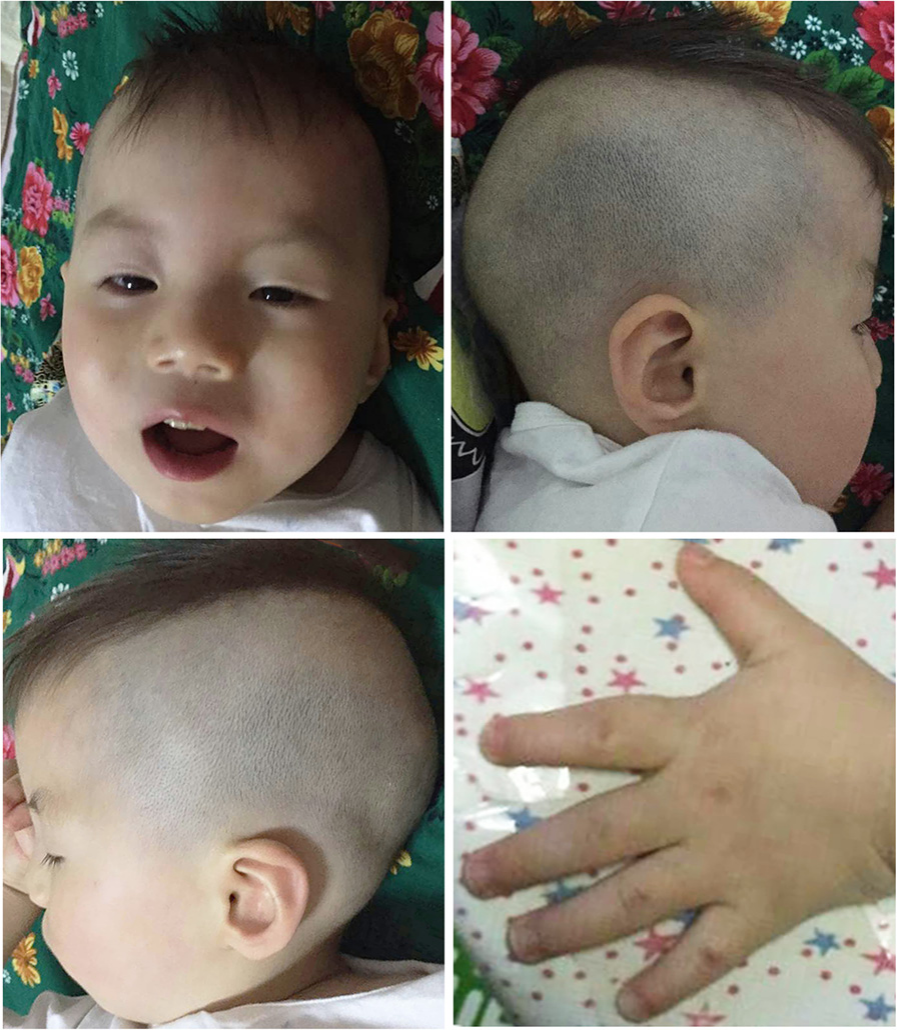
The proband at 2 years 2 months of age. Note scaphocephaly and flat facial profile, auricle dysplasia, low-set and asymmetrical ears, small eyes, flat nose bridge, shallow philtrum, small and open mouth, teeth dysplasia, micrognathia, sparse eyebrows and hair, short hands and broad fingers

The patient was 2 years 2 months old at the time of molecular evaluation. His weight was 12.5 kg (39.7 %), height 86.2 cm (17.9 %) and head circumference 48.5 cm (46.3 %). He demonstrated catch-up growth but hypotonia persisted. His voice was low and he cried weakly. His receptive language was relative normal but he used body language to communicate. His cognitive competence was lower than his peers. On physical examination, his distinctive facial features included scaphocephaly, flat facial profile, auricle dysplasia, low-set and asymmetrical ears, small eyes, flat nose bridge, shallow philtrum, small mouth, teeth dysplasia, micrognathia, sparse eyebrows and hair. He had short hands and broad fingers (Fig. 1). Echocardiography revealed a small atrial septal defect. No genitourinary anomalies was noticed except for small testes. He had chronic and mild to moderate constipation, but no intestinal blockage and enlargement of the colon, and was not diagnosed with Hirschsprung disease. He always displayed a smiling, open-mouth expression and a happy, sociable demeanor as well as timid behavior. He never presented with epileptic seizures, and EEG was normal. The brain magnetic resonance imaging (MRI) showed normal corpus callosum and no other brain structural abnormalities. No additional abnormalities was noticed.

## Methods

### Chromosomal microarray analysis

Chromosomal microarray analysis was performed for the proband and both parents using Affymetrix Cytoscan HD Array (Affymetrix, USA). Genomic DNA was extracted from peripheral blood using a commercial kit (Qiagen). The labeling and hybridization procedures were performed following manufacturer’s instructions. The raw data of chromosomal microarray was analyzed by Affymetrix Chromosome Analysis Suite Software.

## Results

**Fig 2:**
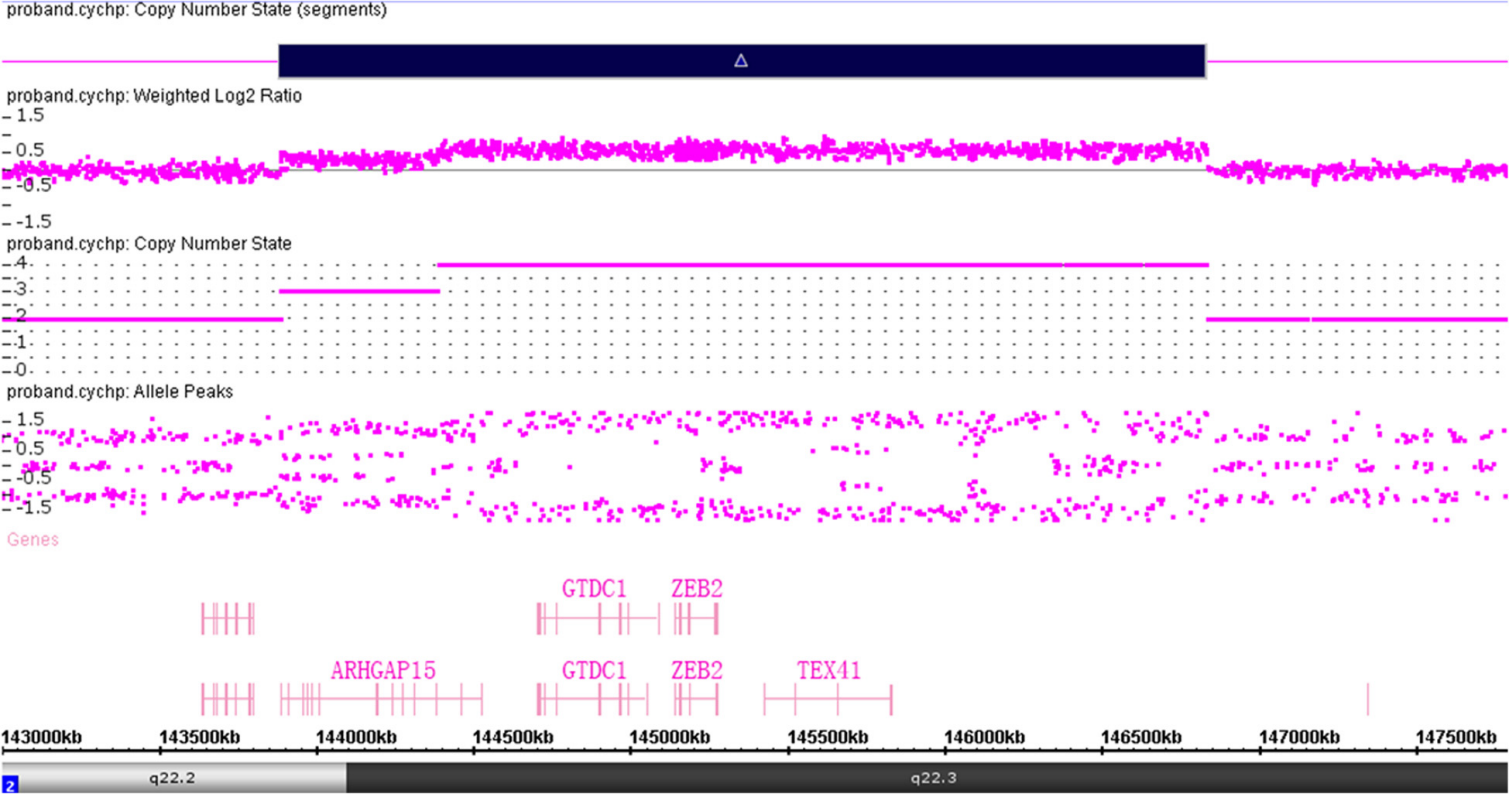
Affymetrix cytoscan HD array analysis including weighted log2 ratio (*upper*), copy number state (*middle*) and allele peaks (*lower*) are shown for chromosome 2. The result shows copy number gain at 2q22.2q22.3 encompassing the entire *ZEB2* gene. The genomic coordinates (hg19): chr2: 143,886,436-146,831,592. The copy number gain region is denoted by a black bar

CMA test revealed a complex gain of copy number at 2q22.2q22.3, which involves a duplication (chr2:143,886,436-144,391,185) and a triplication (chr2:144,391,186-146,831,592) (Fig. 2). Parental tests were normal. Thus, the proband carried a *de novo* copy number variant.

### Discussion

**Fig. 3:**
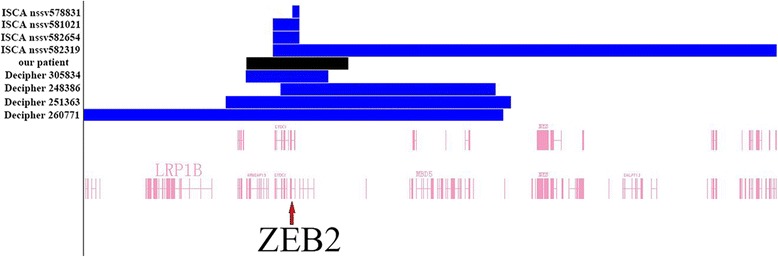
The panel shows a genome view of all duplications or triplication cases (*blue or black colored custom tracks*) relative to the genomic coordinates and RefSeq genes at 2q22.3 region, extracted from Human Genome Build 37 (hg19). Red arrow pinpoints the *ZEB2* gene. Blue: duplication; Black: triplication

*ZEB2* gene mutations or deletions cause Mowat-Wilson syndrome through a haploinsufficiency mechanism, but little is known about the clinical significance of *ZEB2* copy number gain. In this study, we report a 2-year-2-month-old boy with global developmental delay, cognitive impairment, multiple congenital anomalies and behavior problems who carried a *de novo* 2.9 Mb triplication at 2q22.3 involving the entire *ZEB2*, *GTDC1* and *TEX41* genes and part of *ARHGAP15* gene. No other clinical significant CNVs were detected. The patient’s clinical presentation was compared with the typical features of Mowat-Wilson syndrome (Table 1). Some of our patient’s clinical features overlapped with that of Mowat-Wilson syndrome, in particular, severe speech impairment with relative preservation of receptive language, open-mouth appearance and happy demeanor. However, his distinctive facial features were significantly different from that of MWS which included deep-set large and widely spaced eyes, upturned earlobes, saddle nose with rounded nasal tip, pointed chin, flaring eyebrows and elongated face. He had significant intrauterine growth retardation and severe hypotonia whereas he demonstrated postnatal catch-up growth but hypotonia persisted. No triplication at this locus had been reported in literature or described in database. We identified several cases with duplications of *ZEB2* gene in DECIPHER and ISCA databases (Table 2 and Fig. 3). All these duplications were *de novo* except for ones without parental tests and no copy number gain including *ZEB2* gene was reported in the DGV, which strongly suggested a pathogenic nature of these copy number gains.

**Table 1:** Comparison of the clinical features of Mowat-Wilson syndrome and our patient with 2q22.3 triplication involving *ZEB2* gene

Features of MWS	Features of our patient
craniofacial features	
▷ craniosynostosis	craniosynostosis-scaphocephaly
▷ frontal bossing	-
▷ microcephaly	microcephaly at birth, normal at 2 years 2 months
▷ deep-set large and widely spaced eyes	- (small eyes)
▷ large uplifted earlobes with a dimple in the middle	auricle dysplasia, low-set and asymmetrical
▷ a saddle nose with a rounded nasal tip	- (flat nose bridge)
▷ open mouth appearance	+
▷ M-shaped upper lip	-
▷ prominent but narrow chin	- (micrognathia)
▷ large, flaring eyebrows	- (sparse eyebrows and hair)
▷ elongated face	- (flat facial profile)
moderate to severe intellectual disability	mild cognitive impairment
developmental delay	
▷ growth development	+
▷ delayed motor development	+
▷ severe speech impairment with relative preservation of receptive language	+
short stature	IUGR with postnatal catch-up
hypotonia	+
heart defects	+ (small atrial septal defect)
corpus callosum agenesis	-
epilepsy	-
hirschsprung disease	- (mild to moderate constipation)
friendly and happy personalities	+
abnormalities of the urinary tract and genitalia	+ (small testes)
hypospadias	-
eye defects	-
hand anomalies	+ (short hands and broad fingers)
others (skin pigmentary changes, etc.)	-

+ feature present; − feature absent

**Table 2:** Genomic and clinical information of patients with duplication or triplication involving *ZEB2* gene

Patients	Our patient	Decipher 305834	Decipher 248386	Decipher 251363	Decipher 260771	ISCA	ISCA	ISCA
						nssv578831	nssv581021	nssv582319
							nssv582654	
Genomic location (hg19)	chr2:143886436	chr2:143871597	chr2:144872516	chr2:143289932	chr2:139199740	chr2:145219415	chr2:144657717	chr2:144657717
	−146831592	−146250048	−151071321	−151513175	−151305504	−145422833	−145425705	−159178136
Size	2.9 Mb	2.4 Mb	6.2 Mb	8.2 Mb	12.1 Mb	203Kb	768Kb	14.5 Mb
	triplication	duplication	duplication	duplication	duplication	duplication	duplication	duplication
Inheritance	*De novo*	unknown	*De novo*	*De novo*	*De novo*	*De novo*	unknown	unknown
Phenotype	ID, DD, MCA, Behavior problems	ID	Hearing impairment	ID, distinctive facial features, cryptorchidism, macrodontia	ID	seizure	DD, MCA and autism	GDD
Genes involved	*ARHGAP15*, *GTDC1*, *ZEB2*, *TEX41*	*ARHGAP15*, *GTDC1*, *ZEB2*, *TEX41*	*TEX41*, *ACVR2A*, *ORC4*, *MBD5*, *EPC2*, *KIF5C*, *MMADHC*, etc	*ARHGAP15*, *GTDC1*, *ZEB2*, *TEX41*, *ACVR2A*, *ORC4*, *MBD5*, *EPC2*, *KIF5C*, *MMADHC*, etc,	*LRP1B*, *KYNU*, *ARHGAP15*, *GTDC1*, *ZEB2*, *TEX41*, *ACVR2A*, *ORC4*, *MBD5*, *EPC2*, *KIF5C*, *MMADHC*, etc	Part of *ZEB2*	*GTDC1*, *ZEB2*	*GTDC1*, *ZEB2*, *TEX41*, *ACVR2A*, *ORC4*, *MBD5*, *EPC2*, *KIF5C*, *MMADHC*, *NEB*, *CACNB4*, *NR4A2*, *GPD2*, *ACVR1*, etc

Abbreviations: *ID* intellectual disability; *DD* developmental delay; *GDD* Global developmental delay; *MCA* multiple congenital anomalies

There are four genes involved in the copy number gain at 2q22.3 of our patient: *ZEB2*, *GTDC1* and *TEX41* genes are triplicated, part of *ARHGAP15* is duplicated. *ARHGAP15*, a member of the RHO GTPase-activating proteins (GAPs), regulates RHO GTPases (see ARHA; MIM 165390) which regulates diverse biologic processes [15]. *GTDC1* is ubiquitous expressed at relatively high levels in lung, spleen, testis, and peripheral blood leukocytes, suggesting that it may have biochemical functions in these organs [16]. *TEX41* is a non-protein coding gene. Currently, none of the three genes are known to have any clinical significance.

The protein encoded by *ZEB2* gene is a member of δEF1/Zfh-1 family, containing a Smad-binding domain, a homeodomain-like sequence, and two separate clusters of zinc fingers at the amino and carboxy terminals [17]. The ZEB2 protein interacts with SMAD proteins and acts as a transcriptional repressor in response to TGF-β signaling [17]. The SMAD proteins are cytoplasmic mediators that are tightly controlled and play an important role in relaying TGF-ß signals from cell-surface receptors to the nucleus. The TGF-ß family exerts a wide range of biological functions in cell growth, differentiation, apoptosis and development of the embryo. *ZEB2* gene is highly conserved among different species. The homologous alignment at amino acid levels reveals 97 % similarities between human and mouse, and 88 % between human and *Xenopuslaevis*. In addition, these proteins share the same amino acids in the zinc finger domain and certain similarities in their Smad binding domain (SBD). These findings suggest that the protein plays a similar role in vivo.

It was important to note that overexpression of *Xenopus SIP1* (*XSIP1*) induced enlargement of neural tissue in anterior region, and some embryos failed to form eye vesicles and normal head phenotypes. Ectopic expression of *XSIP1* induced anterior neural markers suggesting that *XSIP1* played a role in early neurogenesis [18]. The animal model evidence shows that the *ZEB2* gene is dosage sensitive and its precise regulation and expression is vital to embryonic neural and neural crest development.

Currently several genes have been known to be dosage sensitive genes, such as *MECP2*, *NIPBL* and *NSD1* etc. For example, it is well known that haploinsufficiency of *MECP2* gene typically results in Rett syndrome in females and severe neonatal encephalopathy or lethality in males [19]. Duplications overlapping the entire *MECP2* gene are associated with *MECP2* duplication syndrome characterized by global developmental delay, intellectual disability, autistic features, epilepsy and recurrent infections [20]. Patients with *MECP2* triplications have also been reported with more severe phenotypes [21]. Cornelia de Lange syndrome is a multisystem congenital anomaly disorder and mutations or deletions of *NIPBL* gene is a major cause for this condition [22]. Conversely, *NIPBL* copy number gain is responsible for 5p13 duplication syndrome consisting of developmental delay, learning disability, distinctive facial features and behavior problems [23–25]. Similarly, haploinsufficiency of the *NSD1* gene located on 5q35 is the major cause of Sotos syndrome recognized by intellectual disability, overgrowth, typical facial appearance, behavior problems and seizures [26], whereas reciprocal duplications of Sotos syndrome region overlapping the entire *NSD1* gene present a reverse phenotype including delayed bone age, microcephaly, developmental delay and seizures [27, 28]. We believe more dosage sensitive genes exist in the human genome and are yet to be discovered. Here we provide the first evidence suggesting that *ZEB2* gene is such a dosage sensitive gene similar to the aforementioned genes.

In conclusion, we first report a patient carrying a triplication at 2q22.3 involving the entire *ZEB2* gene who presents overlapping features of Mowat-Wilson syndrome. Based on the clinical evidence from patients with *de novo* copy number gain involving the *ZEB2* gene and the experimental evidence from *Xenopus ZEB2* overexpression model, we propose that *ZEB2* copy number gain is functionally and clinically significant.

### Consent

Written informed consent was obtained from the patient for publication of this Case report and any accompanying images. A copy of the written consent is available for review by the Editor-in-Chief of this journal.
